# Supplementary material for: Tumor Necrosis Factor (TNF) blocking agents are associated with lower risk for Alzheimer’s disease in patients with rheumatoid arthritis and psoriasis
Source: PLoS One. 2020 Mar 23;15(3):e0229819. doi: 10.1371/journal.pone.0229819 (PMC7089534; doi:10.1371/journal.pone.0229819)

**Fig S1**: Adjusted Odds Ratio (AOR) showing the inverse risk association between dementia and TNF blocker (exclude certolizumab pegol and golimumab) or methotrexate compared to the no-drug group adjusting for age, gender, race, and BMI in patients with a diagnosis of rheumatoid arthritis. The analysis excluded patients with both a TNF blocker and methotrexate.


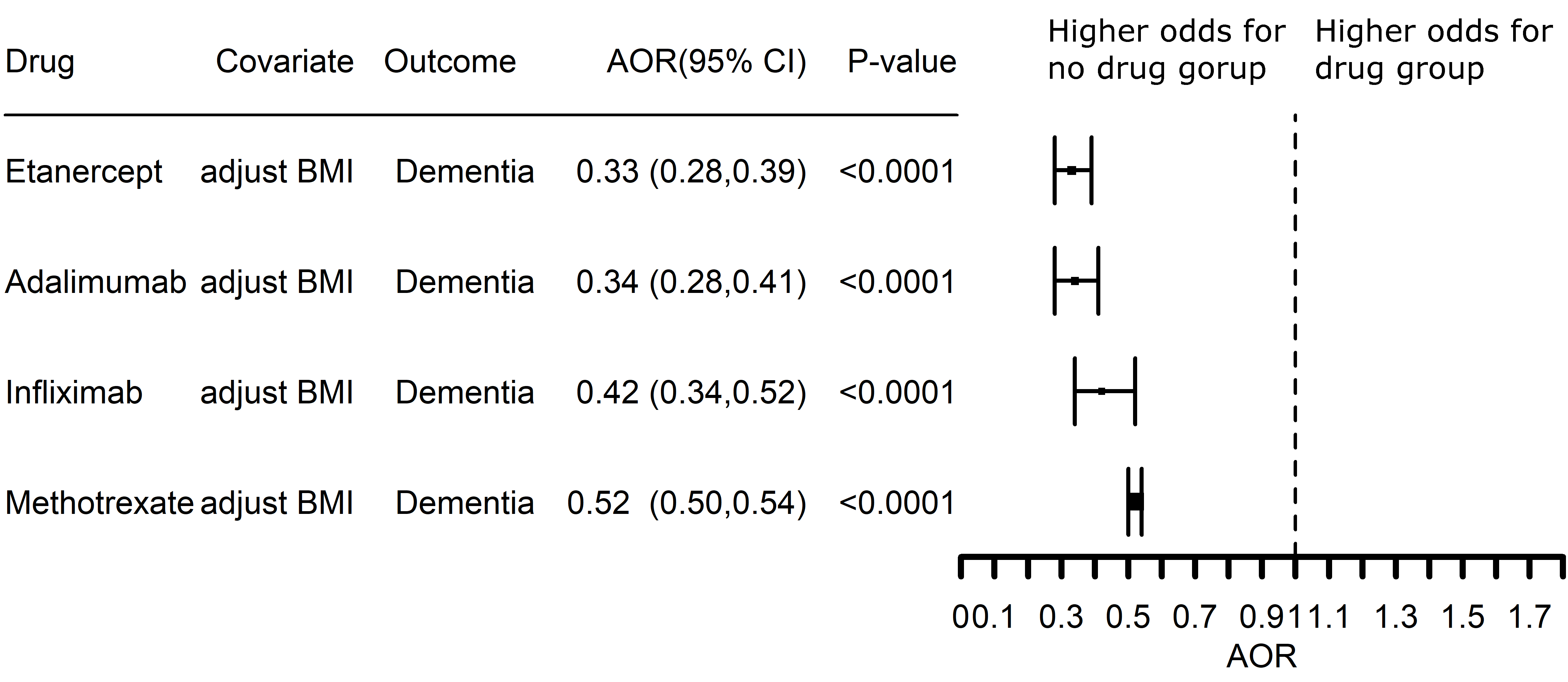

Supplement: S1 Fig — The analysis excluded patients with both a TNF blocker and methotrexate. (DOCX) [file pone.0229819.s001.docx]
