# Supplementary material for: Tumor Necrosis Factor (TNF) blocking agents are associated with lower risk for Alzheimer’s disease in patients with rheumatoid arthritis and psoriasis
Source: PLoS One. 2020 Mar 23;15(3):e0229819. doi: 10.1371/journal.pone.0229819 (PMC7089534; doi:10.1371/journal.pone.0229819)

**Fig S3**: Adjusted Odds Ratio (AOR) showing the inverse risk association between dementia and TNF blocker or methotrexate compared to the no-drug group adjusting for age, gender, and race in patients with a diagnosis of rheumatoid arthritis who are also current non-drinker of alcohol.


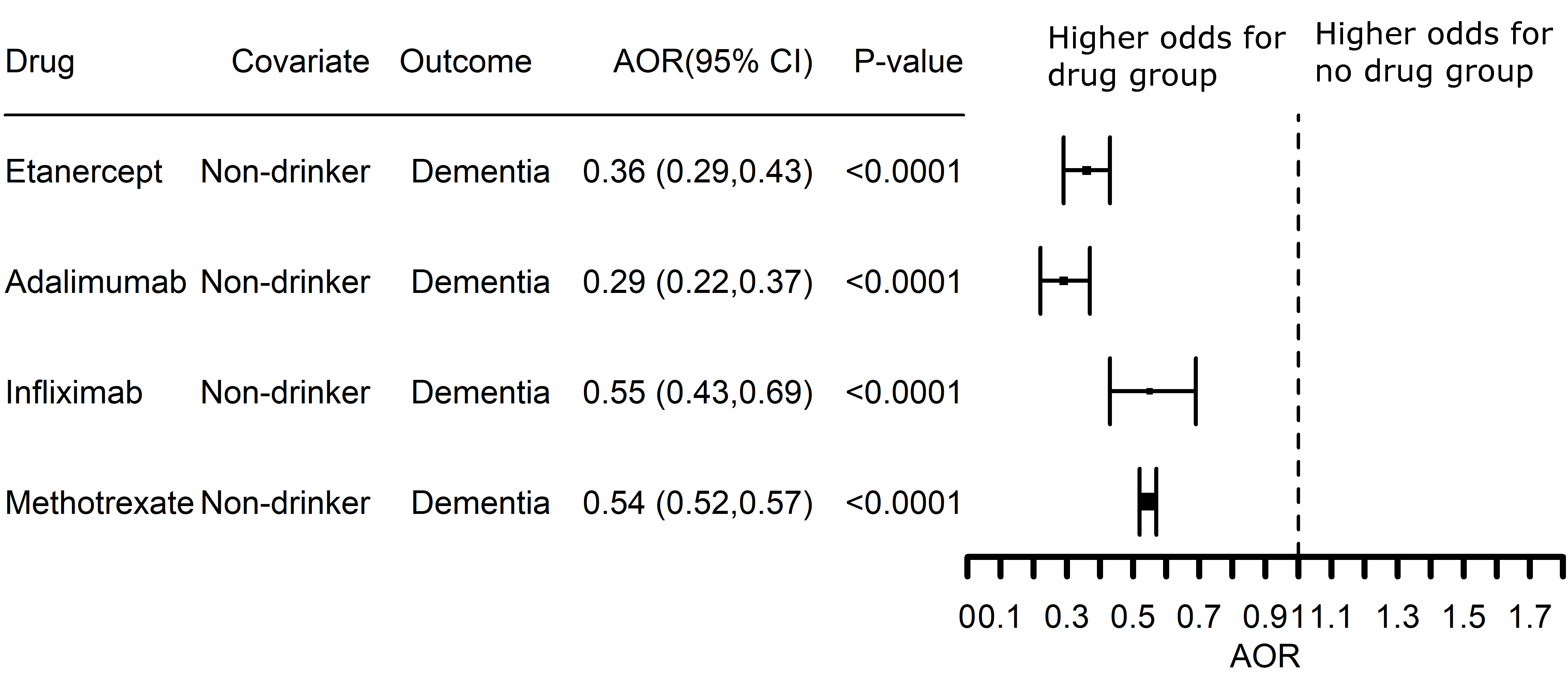

Supplement: S3 Fig — (DOCX) [file pone.0229819.s003.docx]
