# Supplementary material for: Tumor Necrosis Factor (TNF) blocking agents are associated with lower risk for Alzheimer’s disease in patients with rheumatoid arthritis and psoriasis
Source: PLoS One. 2020 Mar 23;15(3):e0229819. doi: 10.1371/journal.pone.0229819 (PMC7089534; doi:10.1371/journal.pone.0229819)
Supplement: S17 Table — (DOCX) [file pone.0229819.s023.docx]

**Table S17**: SNOMED-CT codes of diseases investigated in the study.

| **Disease name** | **SNOMED-CT code** |
| --- | --- |
| Alzheimer’s disease | 26929004 |
| Dementia | 52448006 |
| Rheumatoid arthritis | 69896004 |
| Ankylosing spondylitis | 9631008 |
| Psoriasis | 9014002 |
| Psoriatic arthritis | 156370009 |
| Inflammatory bowel disease | 24526004 |
| Ulcerative colitis | 64766004 |
| Crohn’s disease | 34000006 |
