# Supplementary material for: Tumor Necrosis Factor (TNF) blocking agents are associated with lower risk for Alzheimer’s disease in patients with rheumatoid arthritis and psoriasis
Source: PLoS One. 2020 Mar 23;15(3):e0229819. doi: 10.1371/journal.pone.0229819 (PMC7089534; doi:10.1371/journal.pone.0229819)
Supplement: S22 Table — (DOCX) [file pone.0229819.s028.docx]

**Table S22**: Top 50 common diagnoses between infliximab group and no-drug group

| Description | Count No-drug | Proportion No-drug (%) | Count Infliximab | Proportion Infliximab (%) | Proportion ratio |
| --- | --- | --- | --- | --- | --- |
| Traumatic and/or non-traumatic injury of anatomical site | 250150 | 61 | 2110 | 56 | 1.09 |
| Hypertensive disorder, systemic arterial | 277190 | 67 | 2340 | 62 | 1.08 |
| Disorder of blood vessel | 246470 | 60 | 2100 | 56 | 1.07 |
| Traumatic AND/OR non-traumatic injury | 260290 | 63 | 2230 | 59 | 1.07 |
| Disorder of the genitourinary system | 261830 | 64 | 2270 | 60 | 1.07 |
| Metabolic disease | 301360 | 73 | 2620 | 69 | 1.06 |
| Disorder of thorax | 300150 | 73 | 2620 | 69 | 1.06 |
| Acute disease | 247350 | 60 | 2160 | 57 | 1.05 |
| Disorder of thoracic segment of trunk | 305240 | 74 | 2690 | 71 | 1.04 |
| Disorder of cardiovascular system | 339240 | 82 | 2970 | 79 | 1.04 |
| Essential hypertension | 257390 | 63 | 2290 | 61 | 1.03 |
| Infectious disease | 258710 | 63 | 2320 | 62 | 1.02 |
| Disorder of head | 284740 | 69 | 2560 | 68 | 1.01 |
| Disorder of soft tissue | 348080 | 85 | 3210 | 85 | 1.00 |
| Disorder of respiratory system | 299470 | 73 | 2740 | 73 | 1.00 |
| Disorder of trunk | 367110 | 89 | 3400 | 90 | 0.99 |
| Disorder of abdomen | 315850 | 77 | 2990 | 79 | 0.97 |
| Finding with explicit context | 282170 | 69 | 2660 | 71 | 0.97 |
| Disorder of gastrointestinal tract | 278390 | 68 | 2650 | 70 | 0.97 |
| Disorder of abdominal segment of trunk | 325210 | 79 | 3080 | 82 | 0.96 |
| Disorder of digestive tract | 299900 | 73 | 2870 | 76 | 0.96 |
| Degenerative disorder | 290980 | 71 | 2800 | 74 | 0.96 |
| Soft tissue lesion | 263080 | 64 | 2540 | 67 | 0.96 |
| Disorder of digestive system | 309880 | 75 | 2970 | 79 | 0.95 |
| Chronic disease | 225420 | 55 | 2180 | 58 | 0.95 |
| Disorder of digestive organ | 292850 | 71 | 2840 | 75 | 0.95 |
| Disorder of connective tissue | 283230 | 69 | 2760 | 73 | 0.95 |
| Disorder of extremity | 278390 | 68 | 2720 | 72 | 0.94 |
| Situation with explicit context | 321660 | 78 | 3130 | 83 | 0.94 |
| Disorder of upper digestive tract | 252850 | 61 | 2490 | 66 | 0.92 |
| Osteoarthritis | 239630 | 58 | 2360 | 63 | 0.92 |
| Degenerative disorder of musculoskeletal system | 239650 | 58 | 2360 | 63 | 0.92 |
| Disorder of lower extremity | 231190 | 56 | 2300 | 61 | 0.92 |
| Drug-related disorder | 276660 | 67 | 2790 | 74 | 0.91 |
| Musculoskeletal and connective tissue disorder | 252610 | 61 | 2550 | 68 | 0.9 |
| Procedure with explicit context | 228280 | 56 | 2380 | 63 | 0.89 |
| Disorder of skeletal system | 227020 | 55 | 2370 | 63 | 0.87 |
| Allergic condition | 254770 | 62 | 2710 | 72 | 0.86 |
| Allergic disposition | 237880 | 58 | 2580 | 68 | 0.85 |
| Allergy to substance | 234470 | 57 | 2540 | 67 | 0.85 |
| Hypersensitivity condition | 266210 | 65 | 2900 | 77 | 0.84 |
| Propensity to adverse reactions | 239310 | 58 | 2590 | 69 | 0.84 |
| Hypersensitivity disposition | 239300 | 58 | 2590 | 69 | 0.84 |
| Propensity to adverse reactions to substance | 235980 | 57 | 2560 | 68 | 0.84 |
