# Supplementary material for: Tumor Necrosis Factor (TNF) blocking agents are associated with lower risk for Alzheimer’s disease in patients with rheumatoid arthritis and psoriasis
Source: PLoS One. 2020 Mar 23;15(3):e0229819. doi: 10.1371/journal.pone.0229819 (PMC7089534; doi:10.1371/journal.pone.0229819)
Supplement: S23 Table — (DOCX) [file pone.0229819.s029.docx]

**Table S23**: Top 50 common clinical findings between infliximab group and no drug group

| Description | Count_No-drug | Proportion_No-drug (%) | Count_Infliximab | Proportion_Infliximab (%) | Proportion ratio |
| --- | --- | --- | --- | --- | --- |
| Blood vessel finding | 246880 | 60 | 2100 | 56 | 1.07 |
| Urinary system finding | 236310 | 57 | 2050 | 54 | 1.06 |
| Urogenital finding | 289690 | 70 | 2510 | 67 | 1.04 |
| Cardiovascular finding | 342970 | 83 | 3020 | 80 | 1.04 |
| Finding of region of thorax | 321090 | 78 | 2850 | 76 | 1.03 |
| Finding of upper trunk | 328240 | 80 | 2930 | 78 | 1.03 |
| General body state finding | 247620 | 60 | 2230 | 59 | 1.02 |
| Head finding | 295460 | 72 | 2660 | 71 | 1.01 |
| Neurological finding | 339330 | 83 | 3090 | 82 | 1.01 |
| Finding of trunk structure | 377930 | 92 | 3480 | 92 | 1.00 |
| General finding of soft tissue | 351840 | 86 | 3240 | 86 | 1.00 |
| Finding of head and neck region | 332310 | 81 | 3040 | 81 | 1.00 |
| Sensory nervous system finding | 332030 | 81 | 3070 | 81 | 1.00 |
| Respiratory finding | 314680 | 77 | 2890 | 77 | 1.00 |
| Finding of body region | 401260 | 98 | 3680 | 98 | 1.00 |
| Measurement finding | 228340 | 56 | 2100 | 56 | 1.00 |
| Finding of pelvic region of trunk | 255860 | 62 | 2350 | 62 | 1.00 |
| Pain / sensation finding | 330710 | 80 | 3050 | 81 | 0.99 |
| Pain | 330040 | 80 | 3050 | 81 | 0.99 |
| Pain finding at anatomical site | 324350 | 79 | 3000 | 80 | 0.99 |
| Finding of sensation by site | 326410 | 79 | 3020 | 80 | 0.99 |
| General finding of observation of patient | 319980 | 78 | 2990 | 79 | 0.99 |
| Evaluation finding | 274780 | 67 | 2570 | 68 | 0.99 |
| Pain of truncal structure | 253970 | 62 | 2390 | 63 | 0.98 |
| Measurement finding outside reference range | 223930 | 54 | 2070 | 55 | 0.98 |
| Finding of abdominal segment of trunk | 339240 | 82 | 3180 | 84 | 0.98 |
| Finding of abdomen | 326890 | 79 | 3060 | 81 | 0.98 |
| Clinical history and observation findings | 369650 | 90 | 3520 | 93 | 0.97 |
| Viscus structure finding | 351300 | 85 | 3300 | 88 | 0.97 |
| Musculoskeletal pain | 216080 | 53 | 2070 | 55 | 0.96 |
| Digestive system finding | 316090 | 77 | 3000 | 80 | 0.96 |
| Abdominal organ finding | 314310 | 76 | 2970 | 79 | 0.96 |
| Gastrointestinal tract finding | 288940 | 70 | 2740 | 73 | 0.96 |
| Finding of limb structure | 303430 | 74 | 2930 | 78 | 0.95 |
| Finding of back | 222640 | 54 | 2140 | 57 | 0.95 |
| Finding of lower limb | 253840 | 62 | 2500 | 66 | 0.94 |
| Integumentary system finding | 244050 | 59 | 2360 | 63 | 0.94 |
| Administrative statuses | 270640 | 66 | 2660 | 71 | 0.93 |
| Skin AND/OR mucosa finding | 242010 | 59 | 2420 | 64 | 0.92 |
| Prevention status | 242020 | 59 | 2400 | 64 | 0.92 |
| Mental state, behavior and/or psychosocial function finding | 315240 | 77 | 3170 | 84 | 0.92 |
| Skin finding | 218530 | 53 | 2210 | 59 | 0.9 |
| Behavior finding | 285480 | 69 | 2980 | 79 | 0.87 |
| Health-related behavior finding | 284860 | 69 | 2980 | 79 | 0.87 |
| Finding of tobacco use and exposure | 278430 | 68 | 2930 | 78 | 0.87 |
| Non-smoker | 241980 | 59 | 2610 | 69 | 0.86 |
| Finding of tobacco smoking behavior | 264430 | 64 | 2810 | 75 | 0.85 |
