# Supplementary material for: Tumor Necrosis Factor (TNF) blocking agents are associated with lower risk for Alzheimer’s disease in patients with rheumatoid arthritis and psoriasis
Source: PLoS One. 2020 Mar 23;15(3):e0229819. doi: 10.1371/journal.pone.0229819 (PMC7089534; doi:10.1371/journal.pone.0229819)
Supplement: S24 Table — (DOCX) [file pone.0229819.s030.docx]

**Table S24**: Top 50 common diagnoses between methotrexate group and no-drug group

| Description | Count No-drug | Proportion No-drug (%) | Count Methotrexate | Proportion Methotrexate (%) | Proportion ratio |
| --- | --- | --- | --- | --- | --- |
| Disorder of blood vessel | 246470 | 60 | 49730 | 57 | 1.05 |
| Disorder of the genitourinary system | 261830 | 64 | 52520 | 61 | 1.05 |
| Disorder of gastrointestinal tract | 278390 | 68 | 56440 | 65 | 1.05 |
| Disorder of nervous system | 244820 | 60 | 50180 | 58 | 1.03 |
| Traumatic and/or non-traumatic injury of anatomical site | 250150 | 61 | 51130 | 59 | 1.03 |
| Traumatic AND/OR non-traumatic injury | 260290 | 63 | 53440 | 62 | 1.02 |
| Hypertensive disorder, systemic arterial | 277190 | 67 | 56890 | 66 | 1.02 |
| Disorder of digestive tract | 299900 | 73 | 62250 | 72 | 1.01 |
| Metabolic disease | 301360 | 73 | 62630 | 72 | 1.01 |
| Disorder of thorax | 300150 | 73 | 61960 | 72 | 1.01 |
| Disorder of thoracic segment of trunk | 305240 | 74 | 63060 | 73 | 1.01 |
| Disorder of digestive system | 309880 | 75 | 64240 | 74 | 1.01 |
| Disorder of abdomen | 315850 | 77 | 65680 | 76 | 1.01 |
| Infectious disease | 258710 | 63 | 54140 | 63 | 1.00 |
| Disorder of abdominal segment of trunk | 325210 | 79 | 68020 | 79 | 1.00 |
| Disorder of cardiovascular system | 339240 | 82 | 70960 | 82 | 1.00 |
| Disorder of digestive organ | 292850 | 71 | 61330 | 71 | 1.00 |
| Disorder of respiratory system | 299470 | 73 | 63050 | 73 | 1.00 |
| Disorder of trunk | 367110 | 89 | 77570 | 90 | 0.99 |
| Acute disease | 247350 | 60 | 53200 | 61 | 0.98 |
| Disorder of soft tissue | 348080 | 85 | 75270 | 87 | 0.98 |
| Essential hypertension | 257390 | 63 | 56230 | 65 | 0.97 |
| Disorder of upper digestive tract | 252850 | 61 | 54400 | 63 | 0.97 |
| Disorder of head | 284740 | 69 | 62440 | 72 | 0.96 |
| Finding with explicit context | 282170 | 69 | 63410 | 73 | 0.95 |
| Disorder of connective tissue | 283230 | 69 | 63700 | 74 | 0.93 |
| Drug-related disorder | 276660 | 67 | 62380 | 72 | 0.93 |
| Soft tissue lesion | 263080 | 64 | 59420 | 69 | 0.93 |
| Degenerative disorder | 290980 | 71 | 67060 | 77 | 0.92 |
| Situation with explicit context | 321660 | 78 | 74700 | 86 | 0.91 |
| Musculoskeletal and connective tissue disorder | 252610 | 61 | 59210 | 68 | 0.9 |
| Disorder of extremity | 278390 | 68 | 65640 | 76 | 0.89 |
| Allergic disposition | 237880 | 58 | 56380 | 65 | 0.89 |
| Allergy to substance | 234470 | 57 | 55480 | 64 | 0.89 |
| Hypersensitivity condition | 266210 | 65 | 63510 | 73 | 0.89 |
| Disorder of lower extremity | 231190 | 56 | 54910 | 63 | 0.89 |
| Disorder of skeletal system | 227020 | 55 | 53370 | 62 | 0.89 |
| Allergic condition | 254770 | 62 | 60450 | 70 | 0.89 |
| Propensity to adverse reactions | 239310 | 58 | 56820 | 66 | 0.88 |
| Hypersensitivity disposition | 239300 | 58 | 56820 | 66 | 0.88 |
| Propensity to adverse reactions to substance | 235980 | 57 | 55960 | 65 | 0.88 |
| Degenerative disorder of musculoskeletal system | 239650 | 58 | 57620 | 67 | 0.87 |
| Osteoarthritis | 239630 | 58 | 57610 | 67 | 0.87 |
| Procedure with explicit context | 228280 | 56 | 58880 | 68 | 0.82 |
