# Supplementary material for: Tumor Necrosis Factor (TNF) blocking agents are associated with lower risk for Alzheimer’s disease in patients with rheumatoid arthritis and psoriasis
Source: PLoS One. 2020 Mar 23;15(3):e0229819. doi: 10.1371/journal.pone.0229819 (PMC7089534; doi:10.1371/journal.pone.0229819)
Supplement: S26 Table — (DOCX) [file pone.0229819.s032.docx]

**Table S26**: Counts and proportions (P) of patients with a prescription history of NSAID (or steroids) in each of the drug groups.

| **Drug group** | **Count all** | **Count NSAID** | **P NSAID**  **(%)** | **Count steroids** | **P steroids (%)** |
| --- | --- | --- | --- | --- | --- |
| Etanercept | 8,530 | 7,470 | 88 | 7,270 | 85 |
| Adalimumab | 6,980 | 6,140 | 88 | 6,000 | 86 |
| Infliximab | 3,770 | 3,380 | 90 | 3,300 | 88 |
| Methotrexate | 86,680 | 79,960 | 92 | 78,820 | 91 |
| Comparision (No drug) | 411,800 | 300,850 | 75 | 268,120 | 65 |
