# Supplementary material for: Tumor Necrosis Factor (TNF) blocking agents are associated with lower risk for Alzheimer’s disease in patients with rheumatoid arthritis and psoriasis
Source: PLoS One. 2020 Mar 23;15(3):e0229819. doi: 10.1371/journal.pone.0229819 (PMC7089534; doi:10.1371/journal.pone.0229819)
Supplement: S27 Table — *No-drug group represents patients who did not have prescriptions of any of the TNF blockers or methotrexate. (DOCX) [file pone.0229819.s033.docx]

**Table S27:** Number of patients treated with each of the drugs in each of the inflammatory disease groups. *No-drug group represents patients who did not have prescriptions of any of the TNF blockers or methotrexate.

| Disease group | All | Etanercept | Adalimumab | Infliximab | Methotrexate | No-drug* |
| --- | --- | --- | --- | --- | --- | --- |
| RA | 503,110 | 24,110 | 21,690 | 10,330 | 112,720 | 372,140 |
| AS | 28,490 | 2,130 | 2,590 | 790 | 1,590 | 22,860 |
| Psoriasis | 247,980 | 3,780 | 6,690 | 410 | 9,630 | 230,340 |
| PA | 66,580 | 6,060 | 6,850 | 1,760 | 12,390 | 46,860 |
| IBD | 102,500 | 20 | 40 | 40 | 520 | 101,910 |
| UC | 136,060 | 60 | 3,020 | 4,460 | 1,290 | 128,010 |
| Corhn’s | 160,400 | 70 | 11,560 | 11,960 | 3,670 | 136,190 |
