# Supplementary material for: Tumor Necrosis Factor (TNF) blocking agents are associated with lower risk for Alzheimer’s disease in patients with rheumatoid arthritis and psoriasis
Source: PLoS One. 2020 Mar 23;15(3):e0229819. doi: 10.1371/journal.pone.0229819 (PMC7089534; doi:10.1371/journal.pone.0229819)
Supplement: Attachment — Submitted filename: Response to Reviewers.docx [file pone.0229819.s034.docx]

We greatly appreciate the reviewers’ constructive and insightful comments and suggestions, which have helped us significantly improve the manuscript. Please check our responses in blue. Our manuscript has been revised accordingly.

Reviewer #1: PONE-D-19-26467

This is an interesting study using big data.

Across the manuscript authors report “increased the risk”. The correct description will be “exposure (disease +/- drug) is associated with higher risk for AD”

We have revised the manuscript accordingly.

Authors have selected a limited number of confounders. Confounding bias is one of the most limitations for an observational study like this. Although authors acknowledge this problem in the discussion I miss a deeper external validation for factors used in other studies to find association between inflammatory diseases and AD.

Thank for your comments. We have revised the Study Population (line 124-129, line 147-165), Statistical Analysis (line 186-191, line 205-213), and Results (line 285-288, line 312-314) sections.

For the revised manuscript, we considered additional confounding environmental factors that are known to be associated with both TNF alpha-mediated inflammatory diseases and AD, including smoking, alcohol use, BMI, and obesity. We obtained these confounding factors from references [1]–[8]. The large number of RA (not other inflammatory diseases) patients in the study population allowed us to validate the association of RA with AD after adjusting for the confounding factors of Body Mass Index (BMI) (18.5-24.99 versus >=30), smoking (Non-smoker versus Tobacco user), and alcohol use (Current drinker of alcohol versus Current non-drinker of alcohol), respectively. Since we do not have enough sample size to perform analysis on all possible strata, we did not consider these confounding factors simultaneously. After further adjusting these confounding factors, the association between RA and AD remains statistically significant, but in less degree. The new results are shown in figure 3b.

We further investigated if these environmental confounding factors affected the observed inverse associations of TNF blocking agents (or methotrexate) and the risk of AD. To ensure sufficient sample sizes, we studied RA patients and used the broader diagnosis of dementia as the outcome measure instead of the narrower diagnosis of AD. We adjusted BMI using a method similar to that used for inflammatory diseases. For smoking and alcohol use status, we validated the association between drugs and dementia among patients identified as non-smokers and current non-drinkers of alcohol, respectively, adjusting for age, gender, and race. We do not have enough dementia patients who are tobacco users or alcohol drinkers and who have been prescribed a TNF blocking agent. Figure S1 to S3 show that the significant inverse associations between drugs and dementia remain similar. We also adjusted for steroids use, NSAIDS use, and the diagnosis of hypertension (hypertensive disorder, systemic arterial) as suggested by reviewer #2. The significant inverse associations between drugs and dementia remain similar after controlling for NSAIDS use, steroids use or hypertension (Figure S4 to S6)

**Fig 3b**: Adjusted Odds Ratio (AOR) for a diagnosis of Alzheimer’s disease associated with a diagnosis for rheumatoid arthritis as compared to the non-inflammatory disease group, further adjusting for BMI, smoking status, or alcohol use. The added covariate is presented in column “covariate”. We present both adjusted and stratified AOR for each covariate.

**Fig S1**: Adjusted Odds Ratio (AOR) showing the inverse risk association between dementia and TNF blocker (exclude certolizumab pegol and golimumab) or methotrexate compared to the no-drug group adjusting for age, gender, race, and BMI in patients with a diagnosis of rheumatoid arthritis. The added covariate is presented in column “covariate”. The analysis excluded patients with both a TNF blocker and methotrexate.

**Fig S2**: Adjusted Odds Ratio (AOR) showing the inverse risk association between dementia and TNF blocker or methotrexate compared to the no-drug group adjusting for age, gender, and race in patients with a diagnosis of rheumatoid arthritis who are also non-smokers.

**Fig S3**: Adjusted Odds Ratio (AOR) showing the inverse risk association between dementia and TNF blocker or methotrexate compared to the no-drug group adjusting for age, gender, and race in patients with a diagnosis of rheumatoid arthritis who are also current non-drinker of alcohol.

The authors also acknowledge that the “Cochran-Mantel-Haenszel (CMH) method instead of systematically identifying and controlling for potential confounding factors using a more rigorous multivariable regression method”. My mind concern is that authors are losing statistical power using CMH method, e.g., page 16 lines 350-351 “There were too few patients with Medicaid and Alzheimer’s disease (21,030, 6.2%) or Medicaid and a diagnosis of dementia (84,040, 8.4%) for analysis” This would not be true for a logistic regression analysis. I encourage the authors to repeat the analysis using a multivariable regression approach.

Thank you for your comments and suggestions. We agree that using the Cochran-Mantel-Haenszel (CMH) method would lose statistical power as compared to multivariable logistic regression approaches. EHR data from Explorys cohort discovery are aggregated data instead of individual-patient-level data; therefore, a multivariable logistic regression approach is not suitable. From the database query system, we can build cohorts with any specific characteristics using the following pseudo query *Select patients from Explorys where race == “Caucasian” AND gender==“Female”*. The Explorys cohort discovery platform will return the number of patients in the cohort as the figure shows below:

To adjust for multiple confounding variables, we used CMH to weight average the strata of all possible confounding variables level combinations (given that there are sufficient sample sizes). In the future, we plan to validate this study using patient-level EHR data sources, which we are currently applying for access. Given that the CMH method tends to underestimate the effects, we anticipate that multivariable

regression will yield more significant results.

Abstract: the abstract does not say what analysis what used.

We have revised the abstract (line 28-30) and added: “Adjusted odds ratios (AORs) were estimated using the Cochran-Mantel-Haenszel method and presented with 95% confidence intervals (CIs) and p-values.”

Methods:

• could the authors list SNOMED-CT codes in Appendix in order to allow reproducibility of their work

Thank you for the suggestions. We have listed the SNOMED-CT codes in table S17:

**Table S17**: SNOMED-CT codes of diseases.

| **Disease name** | **SNOMED-CT code** |
| --- | --- |
| Alzheimer’s disease | 26929004 |
| Dementia | 52448006 |
| Rheumatoid arthritis | 69896004 |
| Ankylosing spondylitis | 9631008 |
| Psoriasis | 9014002 |
| Psoriatic arthritis | 156370009 |
| Inflammatory bowel disease | 24526004 |
| Ulcerative colitis | 64766004 |
| Crohn’s disease | 34000006 |

• Could the authors report how was performed the matching, please? Page 7 lines 140-144

We thank the reviewer’s suggestion and have revised the Study Population part (line 141-144) as following:

We matched the diagnosis of RA or psoriasis between each drug group to a comparison (no-drug) group of patients who did not have prescriptions for any TNF blocking agents or methotrexate. The effect of each drug treatment on the risk for Alzheimer’s disease or dementia (including Alzheimer’s disease) was then compared to the no-drug group adjusting for confounding demographic factors including age, gender, and race.

• Insurance as a proxy for socioeconomic status seems to be of difficult interpretation for patients older than 65. Page 8 line 179.

Thank you for comments. Patients > 65 years with low socioeconomic status often receive Medicaid plus Medicare. Therefore, we have revised our analysis and the section (line 202-204) by adjusting Medicaid use instead of Medicare use among patients > 65. There were not enough dementia patients for analysis who used Medicaid and who also were prescribed a TNF blocking agent. Therefore, for patients > 65 years old, the AORs were calculated among patients who did not receive Medicaid by controlling for gender and race. The revised figure 10 shows that the significant inverse associations between drugs and dementia remain similar after adjustment for Medicaid.

**Fig 10**. Crude Odds Ratios (OR) and Adjusted Odds Ratios (AOR) based on insurance status (Medicaid versus no-Medicare, or private versus no-private insurance) in patients with a diagnosis code for dementia and rheumatoid arthritis.

Results

• Could the authors show in a table differences of patients with missing values excluded for analysis in terms of exposures and outcomes, please? And report how missing values were addressed in methods.

Thank you for the comments. We built cohorts by selecting patients with specific characteristics. *(Select patients from Explorys where race == “Caucasian” AND gender==“Female”*), therefore patients with missing values (value of “Null” ) were not included in our study. We assume the missing values shall not be systematically biased against or towards AD or dementia diagnosis. In the future, we plan to validate the study using patient-level EHR data sources, where we can investigate how missing values affect our findings in this study.

• Could the authors move to the discussion page 14 lines 317 to 320 and describe how the current/routine practice is, please?

In normal clinical practice, patients are started on methotrexate and those who fail methotrexate may progress to treatment with a TNF blocking agent. Of those who progress to the TNF blocking agent, 60% will continue on methotrexate and 40% will drop the methotrexate.

Reviewer #2: Overall, this is an important and impressive study, leveraging an extremely powerful data source that covers a substantial portion of the US population electronic health data. The major findings are convincing and conclusions mostly well justified. However, there are some limitations including a fairly simplistic analysis. In general, the study would be strengthened by some additional data on comparisons of treated vs no treatment.

Specific comments and questions are raised below:

The AOR are calculated comparing the reduction in risk for AD/dementia with a given diagnosis (eg, RA, Figure 5) compared to the no-drug group. There may be a selection bias in the severity of disease, insurance, and/or many other factors that contribute to whether or not treatment is administered. Some effort to control these confounders should be attempted, eg., at least using other potential diagnoses and medications and propensity scores. At least providing a Table with comparisons of treated vs untreated would be very helpful.

We thank the reviewer’s comments and suggestions. We have revised the Study Population (line 124-129, line 147-165), Statistical Analysis (line 186-191, line 205-213), and Results (line 285-288, line 312-314) parts.

For this revision, we extracted the top 50 diagnoses and top 50 clinical findings for each drug group and the corresponding no-drug group. The shared disease diagnoses (or clinical findings) between each drug group and the no-drug group are presented in Table S17 to S25. The proportion ratios of diagnoses and clinical findings comparing no drug group versus drug group are close to 1.00.

We then selected the diagnoses and clinical findings with the biggest difference between drug group and its corresponding no-drug group. Among the top 50 diagnoses (Table S17), “Hypertensive disorder, systemic arterial” has the biggest difference between drug and no-drug groups. Among the top 50 clinical findings (Table S18), “Non-smoker” has biggest difference between no-drug and drug group. “Hypertensive disorder, systemic arterial”, and “Non-smoker” are known to be associated with Alzheimer’s disease (or dementia), as suggested elsewhere[3], [9]. We controlled for smoking status as suggested by reviewer #1 (Figure S2) and found that it has no significant effects on the observed inverse risk associations between drug and dementia. Similarly, we controlled for the diagnosis of “Hypertensive disorder, systemic arterial” and examined the associations between drugs and AD/dementia among RA patients. To ensure enough sample sizes, we used a broader diagnosis of dementia as the outcome measure instead of the narrower diagnosis of AD. Figure S4 shows that the inverse associations between drugs and dementia are similar and remain significant among patients with a diagnosis of “Hypertensive disorder, systemic arterial”.

**Table S17**: Top 50 common diagnoses between enternercept group and no-drug group

| Description | Count No- drug | Proportion No- drug (%) | Count Etanercept | Proportion Etanercept (%) | Proportion ratio |
| --- | --- | --- | --- | --- | --- |
| Hypertensive disorder, systemic arterial | 277190 | 67 | 4580 | 54 | 1.24 |
| Traumatic and/or non-traumatic injury of anatomical site | 250150 | 61 | 4260 | 50 | 1.22 |
| Metabolic disease | 301360 | 73 | 5210 | 61 | 1.2 |
| Traumatic AND/OR non-traumatic injury | 260290 | 63 | 4480 | 53 | 1.19 |
| Essential hypertension | 257390 | 63 | 4500 | 53 | 1.19 |
| Disorder of the genitourinary system | 261830 | 64 | 4610 | 54 | 1.19 |
| Disorder of thorax | 300150 | 73 | 5250 | 62 | 1.18 |
| Disorder of thoracic segment of trunk | 305240 | 74 | 5380 | 63 | 1.17 |
| Disorder of gastrointestinal tract | 278390 | 68 | 4960 | 58 | 1.17 |
| Disorder of cardiovascular system | 339240 | 82 | 6140 | 72 | 1.14 |
| Acute disease | 247350 | 60 | 4610 | 54 | 1.11 |
| Disorder of digestive tract | 299900 | 73 | 5620 | 66 | 1.11 |
| Infectious disease | 258710 | 63 | 4870 | 57 | 1.11 |
| Disorder of abdomen | 315850 | 77 | 6040 | 71 | 1.08 |
| Disorder of abdominal segment of trunk | 325210 | 79 | 6260 | 73 | 1.08 |
| Chronic disease | 225420 | 55 | 4310 | 51 | 1.08 |
| Disorder of digestive organ | 292850 | 71 | 5650 | 66 | 1.08 |
| Disorder of respiratory system | 299470 | 73 | 5810 | 68 | 1.07 |
| Disorder of digestive system | 309880 | 75 | 5930 | 70 | 1.07 |
| Disorder of upper digestive tract | 252850 | 61 | 4870 | 57 | 1.07 |
| Disorder of connective tissue | 283230 | 69 | 5560 | 65 | 1.06 |
| Disorder of head | 284740 | 69 | 5520 | 65 | 1.06 |
| Degenerative disorder | 290980 | 71 | 5670 | 67 | 1.06 |
| Degenerative disorder of musculoskeletal system | 239650 | 58 | 4700 | 55 | 1.05 |
| Osteoarthritis | 239630 | 58 | 4700 | 55 | 1.05 |
| Disorder of soft tissue | 348080 | 85 | 7000 | 82 | 1.04 |
| Disorder of trunk | 367110 | 89 | 7330 | 86 | 1.03 |
| Soft tissue lesion | 263080 | 64 | 5270 | 62 | 1.03 |
| Disorder of skeletal system | 227020 | 55 | 4600 | 54 | 1.02 |
| Musculoskeletal and connective tissue disorder | 252610 | 61 | 5110 | 60 | 1.02 |
| Finding with explicit context | 282170 | 69 | 5790 | 68 | 1.01 |
| Disorder of lower extremity | 231190 | 56 | 4790 | 56 | 1.00 |
| Disorder of extremity | 278390 | 68 | 5870 | 69 | 0.99 |
| Drug-related disorder | 276660 | 67 | 5900 | 69 | 0.97 |
| Situation with explicit context | 321660 | 78 | 6950 | 82 | 0.95 |
| Procedure with explicit context | 228280 | 56 | 5090 | 60 | 0.93 |
| Propensity to adverse reactions | 239310 | 58 | 5470 | 64 | 0.91 |
| Hypersensitivity disposition | 239300 | 58 | 5470 | 64 | 0.91 |
| Allergic disposition | 237880 | 58 | 5430 | 64 | 0.91 |
| Propensity to adverse reactions to substance | 235980 | 57 | 5380 | 63 | 0.9 |
| Allergy to substance | 234470 | 57 | 5350 | 63 | 0.9 |
| Allergic condition | 254770 | 62 | 5850 | 69 | 0.9 |
| Hypersensitivity condition | 266210 | 65 | 6270 | 74 | 0.88 |

**Table S18**: Top 50 common clinical findings between etanercept group and no-drug group

| Description | Count No-drug | Proportion No-drug (%) | Count Etanercept | Proportion Etanercept(%) | Proportion ratio |
| --- | --- | --- | --- | --- | --- |
| Gastrointestinal tract finding | 288940 | 70 | 5230 | 61 | 1.15 |
| Measurement finding | 228340 | 56 | 4200 | 49 | 1.14 |
| Cardiovascular finding | 342970 | 83 | 6260 | 73 | 1.14 |
| Finding of region of thorax | 321090 | 78 | 5860 | 69 | 1.13 |
| Finding of upper trunk | 328240 | 80 | 6060 | 71 | 1.13 |
| Urogenital finding | 289690 | 70 | 5380 | 63 | 1.11 |
| General body state finding | 247620 | 60 | 4630 | 54 | 1.11 |
| Pain of truncal structure | 253970 | 62 | 4830 | 57 | 1.09 |
| Abdominal organ finding | 314310 | 76 | 5980 | 70 | 1.09 |
| Digestive system finding | 316090 | 77 | 6080 | 71 | 1.08 |
| Head finding | 295460 | 72 | 5730 | 67 | 1.07 |
| Finding of abdomen | 326890 | 79 | 6320 | 74 | 1.07 |
| Evaluation finding | 274780 | 67 | 5410 | 63 | 1.06 |
| Viscus structure finding | 351300 | 85 | 6810 | 80 | 1.06 |
| Respiratory finding | 314680 | 77 | 6180 | 73 | 1.05 |
| Finding of abdominal segment of trunk | 339240 | 82 | 6640 | 78 | 1.05 |
| Finding of head and neck region | 332310 | 81 | 6650 | 78 | 1.04 |
| Neurological finding | 339330 | 83 | 6820 | 80 | 1.04 |
| General finding of soft tissue | 351840 | 86 | 7080 | 83 | 1.04 |
| Finding of pelvic region of trunk | 255860 | 62 | 5080 | 60 | 1.03 |
| General finding of observation of patient | 319980 | 78 | 6440 | 76 | 1.03 |
| Sensory nervous system finding | 332030 | 81 | 6770 | 79 | 1.03 |
| Finding of trunk structure | 377930 | 92 | 7630 | 90 | 1.02 |
| Finding of sensation by site | 326410 | 79 | 6680 | 78 | 1.01 |
| Pain finding at anatomical site | 324350 | 79 | 6640 | 78 | 1.01 |
| Pain | 330040 | 80 | 6730 | 79 | 1.01 |
| Pain / sensation finding | 330710 | 80 | 6750 | 79 | 1.01 |
| Finding of body region | 401260 | 98 | 8310 | 98 | 1 |
| Finding of limb structure | 303430 | 74 | 6390 | 75 | 0.99 |
| Finding of lower limb | 253840 | 62 | 5330 | 63 | 0.98 |
| Integumentary system finding | 244050 | 59 | 5100 | 60 | 0.98 |
| Musculoskeletal pain | 216080 | 53 | 4630 | 54 | 0.98 |
| Administrative statuses | 270640 | 66 | 5910 | 69 | 0.96 |
| Clinical history and observation findings | 369650 | 90 | 8080 | 95 | 0.95 |
| Skin finding | 218530 | 53 | 4780 | 56 | 0.95 |
| Prevention status | 242020 | 59 | 5340 | 63 | 0.94 |
| Skin AND/OR mucosa finding | 242010 | 59 | 5360 | 63 | 0.94 |
| Mental state, behavior and/or psychosocial function finding | 315240 | 77 | 7550 | 89 | 0.87 |
| Behavior finding | 285480 | 69 | 7280 | 85 | 0.81 |
| Health-related behavior finding | 284860 | 69 | 7280 | 85 | 0.81 |
| Finding of tobacco smoking behavior | 264430 | 64 | 6770 | 79 | 0.81 |
| Finding of tobacco use and exposure | 278430 | 68 | 7180 | 84 | 0.81 |
| Non-smoker | 241980 | 59 | 6210 | 73 | 0.81 |

**Fig S4**: Adjusted Odds Ratio (AOR) showing the inverse risk association between dementia and TNF blocker or methotrexate compared to the no-drug group adjusting for age, gender, race in patients with a diagnosis of rheumatoid arthritis and “hypertensive disorder, systemic arterial”. The analysis excluded patients with both a TNF blocker and methotrexate.

**Fig S1**: Adjusted Odds Ratio (AOR) showing the inverse risk association between dementia and TNF blocker (exclude certolizumab pegol and golimumab) or methotrexate compared to the no-drug group adjusting for age, gender, race, and BMI in patients with a diagnosis of rheumatoid arthritis. The analysis excluded patients with both a TNF blocker and methotrexate.

**Fig S2**: Adjusted Odds Ratio (AOR) showing the inverse risk association between dementia and TNF blocker or methotrexate compared to the no-drug group adjusting for age, gender, and race in patients with a diagnosis of rheumatoid arthritis who are also non-smokers.

**Fig S3**: Adjusted Odds Ratio (AOR) showing the inverse risk association between dementia and TNF blocker or methotrexate compared to the no-drug group adjusting for age, gender, and race in patients with a diagnosis of rheumatoid arthritis who are also current non-drinker of alcohol.

Similarly, it is not clear if the “no-drug” group included patients with inflammatory conditions treated with other medications such as NSAIDS, or steroids. As pointed out in the discussion, “NSAIDs suppress prostaglandin synthesis by inhibiting the cyclooxygenase enzymes COX-1 and COX-2, and thereby reduce immune cell activation, but may increase TNF production [47, 55]”. Given this possibility, NSAID use may be a confound that should be addressed.

We thank the reviewer for bringing up this idea. We have revised the Study Population (line 161-165), Statistical Analysis (line 209-213), and Results (line 312-314) parts.

In the revised manuscript, we have listed the proportions of patients who used NSAIDs or steroids among each drug group and the no-drug group. Table S26 shows the proportion of patients who used NSAIDs or steroids was higher for drug groups comparing with the no-drug group. There were not enough dementia patients who have been prescribed anti-TNF drugs ***but not*** prescribed NSAIDs or steroids. Thus, we compared patients received each of the TNF blocking agent (or methotrexate) plus NSAIDs (or steroid) with a group of patients who received only NSAIDs (or steroids) but no TNF blocking agents or methotrexate. Figure S4 to S5 show that the significant inverse associations between drugs and dementia remain similar after controlling for NSAIDs and steroids.

**Table S26**: Counts and proportions (P) of patients with a prescription history of NSAID (or steroids) in each of the drug groups.

| **Drug group** | **Count all** | **Count NSAIDs** | **P NSAIDs**  **(%)** | **Count steroids** | **P steroids (%)** |
| --- | --- | --- | --- | --- | --- |
| Etanercept | 8,530 | 7,470 | 88 | 7,270 | 85 |
| Adalimumab | 6,980 | 6,140 | 88 | 6,000 | 86 |
| Infliximab | 3,770 | 3,380 | 90 | 3,300 | 88 |
| Methotrexate | 86,680 | 79,960 | 92 | 78,820 | 91 |
| Comparision (No drug) | 411,800 | 300,850 | 75 | 268,120 | 65 |

**Fig S5**: Adjusted Odds Ratio (AOR) showing the inverse risk association between dementia and TNF blocker or methotrexate compared to the no-drug group adjusting for age, gender, and race in patients with a diagnosis of rheumatoid arthritis who also prescribed NSAID.

**Fig S6**: Adjusted Odds Ratio (AOR) showing the inverse risk association between dementia and TNF blocker or methotrexate compared to the no-drug group adjusting for age, gender, and race in patients with a diagnosis of rheumatoid arthritis who also prescribed steriods.

The manuscript states: “Systemic inflammatory disease is a substantial risk factor for Alzheimer’s disease (Figure 3). Greatest risk is associated with inflammatory bowel disease and Crohn’s disease followed by rheumatoid arthritis, ulcerative colitis (as a subcategory of inflammatory bowel disease), ankylosing spondylitis and psoriasis.” How many of those with each diagnosis were treated? This information may be buried in table 1 but it would help if there were explicit numbers provided in the figure or table. Were these numbers that were used to derive the OR based only on the untreated numbers of patients (eg. RA) or all patients with each diagnosis regardless of treatment condition?

All of the patients in each of the inflammatory disease groups were included to compute the adjusted odds ratios (AORs). We list the number of patients that were treated with each of the drugs in each of the inflammatory disease group in Table S27.

**Table S27:** Number of patients treated with each of the drugs in each of the inflammatory disease groups. *No-drug group represents patients who did not have prescriptions of any of the TNF blockers or methotrexate.

| Disease group | All | Etanercept | Adalimumab | Infliximab | Methotrexate | No-drug* |
| --- | --- | --- | --- | --- | --- | --- |
| RA | 503,110 | 24,110 | 21,690 | 10,330 | 112,720 | 372,140 |
| AS | 28,490 | 2,130 | 2,590 | 790 | 1,590 | 22,860 |
| Psoriasis | 247,980 | 3,780 | 6,690 | 410 | 9,630 | 230,340 |
| PA | 66,580 | 6,060 | 6,850 | 1,760 | 12,390 | 46,860 |
| IBD | 102,500 | 20 | 40 | 40 | 520 | 101,910 |
| UC | 136,060 | 60 | 3,020 | 4,460 | 1,290 | 128,010 |
| Corhn’s | 160,400 | 70 | 11,560 | 11,960 | 3,670 | 136,190 |

“Indeed, the risk for Alzheimer’s disease was reduced below the general population risk in rheumatoid arthritis patients treated with adalimumab (AOR 0.62 (0.43 – 0.89) and in psoriasis patients treated with etanercept (AOR 0.58 (0.37 – 0.90) or adalimumab (AOR 0.48 (0.23 – 0.88), Figure 11.” However, the same figure shows that for RA, the adalimumab or infliximab treatments still yielded higher adjusted odds ratios (AOR >1) compared to the general population. This should be discussed, as not all TNF blocking agents reduce the risk for Alzheimer’s disease below the risk in the general population in the absence of inflammatory disease.

Since RA is associated with increased risks of AD and dementia (figure 3 and figure 4) and our observed inverse risk associations between TNF blocking agents and AD/dementia are among patients with RA. Therefore, TNF blocking agents did not reduce the risk for AD and dementia for RA patients below the general population given that RA is a risk factor or AD/dementia.

Comparison of AD risk vs another CNS disorder that might not be expected to involve systemic inflammation may be a useful control. For example, consider stroke or migraine. These or other “control” comparisons would bolster confidence in the findings from this database.

We thank the reviewer for bringing up this idea.

First, given that systemic inflammation affects many organs, it is very difficult to find and prove that certain other CNS disorders definitely have no relationships with systemic inflammation. Literature shows that systemic inflammation plays roles in stroke or migraine[10], [11].

Second, we used well-established (and straightforward) statistic methods in this study. Our findings between AD risk and systemic inflammatory diseases confirmed published studies and are served as a positive control (for both statistical methods and the EHR data) for our findings between AD risks and TNF alpha blockers.

Third, the goal of this study is to investigate the risk associations between TNF blockers and AD/dementia, therefore, these “control comparison” between AD risk and other control diseases shall not affect our main conclusion.

I could not find the time windows that constrained the analyses. How long were records available – in years? That is, in calculating the OR for RA, how many years minimum and maximum were allowed prior to outcome of Alzheimer? Ditto for other diseases.

Thank you for pointing this out. EHR from Explorys cohort discovery are aggregated data instead of individual-patient-level data. Patient-level information like exact age at diagnosis, duration of treatment, or years of follow up are not accessible. Therefore, we were not able to constrain the time windows in this study. We are currently applying for EHR sources with patient-level data. In the future, we plan to validate the study using patient-level EHR data sources.

The analyses do not seem to adjust for multiple comparisons; given this large sample size it is critical to do so.

We thank the reviewer for bringing this up. We tested 4 drugs and 7 diseases in the study. We assume that correcting for multiple comparisons shall not influence the conclusion since the number of hypotheses is small. For example, we validated the results in figure 4 and figure 5 by adjusting for the multiple-comparison using Bonferroni correction. The significance threshold is 0.0071 and 0.0125 for figure 4 and figure 5 after the adjustment. Since all significant p-values were less than 0.0001, all the associations remain significant after correcting for multiple comparisons.

How about other dementia subtypes such as DLB and FTD? Dx are also usually stratified for EOAD (presenile dementia).

Thank you for the comments. Since TNF inhibitors are a rare exposure to dementia, we only have enough sample size to study the association between TNF inhibitors and Alzheimer’s disease and the broader type of dementia. We don’t have enough sample sizes for the rare subtypes of dementia (in patients with diagnosis of RA and also on a TNF inhibitor)

Medication use was defined as “taking a medication” if at least one outpatient prescription was identified. To obviate one-time use, it would be helpful to see if the effects are altered by defining as at least 2 prescriptions to help insure adequate drug exposure.

Thank you for the comments. As we discussed in the question related to the time window, EHR from Explorys cohort discovery are aggregated data instead of individual-patient-level data. Patient-level information including times of prescriptions are not available. The effect prescription times can be validated using the patient-level EHR data which we are currently applying for. In addition, RA is a chronic and incurable disease, and we assume that patients are likely not one-time use for these drugs.

The discussion addresses the few previous clinical trials of TNF antagonist therapies in symptomatic AD. These trials all seem to be based on clinical benefit as outcomes, but the retrospective study in this paper is examining AD/dementia as an outcome. It should be pointed out that these findings suggest the potential neuroprotective effects of anti-inflammatory treatments involving TNF signaling, rather than clinical symptomatic benefits.

We have revised the discussion part (line 368-377) as follows:

Etanercept previously was evaluated for symptomatic benefit in patients with mild-to-moderate Alzheimer’s disease in a small, placebo-controlled study enrolling 41 subjects. End points in the study assessed improvement in cognitive function and activities of daily living. There was a trend for etanercept benefit at 24 weeks of treatment across multiple assessments. In contrast, our retrospective case study which used conversion to Alzheimer’s disease or dementia as an outcome measure suggests the potential neuroprotective benefit of interrupting TNF signaling between the periphery and the brain. This benefit could translate to other neurodegenerative diseases as well as to psychiatric disorders. A previous study of Parkinson’s disease in patients with co-morbid inflammatory bowel disease reported that TNF blocking agents were associated with decreased incidence of Parkinson’s disease, while therapeutic benefit was reported earlier for reducing risk of major depression in a pivotal, Phase 3 clinical trial of etanercept for the treatment of psoriasis.

Reviewer #3: This is a very well done manuscript on an important topic. The writing is clear and not ambiguous. The conclusions support the concept of inflammation in Alzheimer's disease, which is a basic but little emphasized topic.

We appreciate the reviewers’ positive comments on the manuscript.

**References**

[1]	A. K. Piazza-Gardner, T. J. B. Gaffud, and A. E. Barry, “The impact of alcohol on Alzheimer’s disease: a systematic review,” *Aging Ment. Health*, vol. 17, no. 2, pp. 133–146, 2013.

[2]	L. A. Profenno, A. P. Porsteinsson, and S. V Faraone, “Meta-analysis of Alzheimer’s disease risk with obesity, diabetes, and related disorders,” *Biol. Psychiatry*, vol. 67, no. 6, pp. 505–512, 2010.

[3]	M. Baumgart, H. M. Snyder, M. C. Carrillo, S. Fazio, H. Kim, and H. Johns, “Summary of the evidence on modifiable risk factors for cognitive decline and dementia: a population-based perspective,” *Alzheimer’s Dement.*, vol. 11, no. 6, pp. 718–726, 2015.

[4]	D. Di Giuseppe, L. Alfredsson, M. Bottai, J. Askling, and A. Wolk, “Long term alcohol intake and risk of rheumatoid arthritis in women: a population based cohort study,” *Bmj*, vol. 345, p. e4230, 2012.

[5]	B. Lu, D. H. Solomon, K. H. Costenbader, and E. W. Karlson, “Alcohol consumption and risk of incident rheumatoid arthritis in women: a prospective study,” *Arthritis Rheumatol.*, vol. 66, no. 8, pp. 1998–2005, 2014.

[6]	B. Qin *et al.*, “Body mass index and the risk of rheumatoid arthritis: a systematic review and dose-response meta-analysis,” *Arthritis Res. Ther.*, vol. 17, no. 1, p. 86, 2015.

[7]	D. Sugiyama *et al.*, “Impact of smoking as a risk factor for developing rheumatoid arthritis: a meta-analysis of observational studies,” *Ann. Rheum. Dis.*, vol. 69, no. 01, pp. 70–81, 2010.

[8]	L. Klareskog *et al.*, “A new model for an etiology of rheumatoid arthritis: smoking may trigger HLA–DR (shared epitope)–restricted immune reactions to autoantigens modified by citrullination,” *Arthritis Rheum. Off. J. Am. Coll. Rheumatol.*, vol. 54, no. 1, pp. 38–46, 2006.

[9]	P. Gilsanz *et al.*, “Female sex, early-onset hypertension, and risk of dementia,” *Neurology*, vol. 89, no. 18, pp. 1886–1893, 2017.

[10]	Z. Nadareishvili, K. Michaud, J. M. Hallenbeck, and F. Wolfe, “Cardiovascular, rheumatologic, and pharmacologic predictors of stroke in patients with rheumatoid arthritis: a nested, case–control study,” *Arthritis Care Res. (Hoboken).*, vol. 59, no. 8, pp. 1090–1096, 2008.

[11]	Y.-C. Wang, Y.-P. Huang, M.-T. Wang, H.-I. Wang, and S.-L. Pan, “Increased risk of rheumatoid arthritis in patients with migraine: a population-based, propensity score-matched cohort study,” *Rheumatol. Int.*, vol. 37, no. 2, pp. 273–279, 2017.

**Table S20:** Top 50 common diagnoses between adalimumab group and no-drug group

| Description | Count_No-drug | Proportion No-drug (%) | Count Adalimumab | Proportion Adalimumab (%) | Proportion ratio |
| --- | --- | --- | --- | --- | --- |
| Hypertensive disorder, systemic arterial | 277190 | 67 | 3860 | 55 | 1.22 |
| Disorder of the genitourinary system | 261830 | 64 | 3840 | 55 | 1.16 |
| Traumatic and/or non-traumatic injury of anatomical site | 250150 | 61 | 3670 | 53 | 1.15 |
| Traumatic AND/OR non-traumatic injury | 260290 | 63 | 3840 | 55 | 1.15 |
| Essential hypertension | 257390 | 63 | 3800 | 55 | 1.15 |
| Disorder of thorax | 300150 | 73 | 4460 | 64 | 1.14 |
| Metabolic disease | 301360 | 73 | 4510 | 65 | 1.12 |
| Disorder of thoracic segment of trunk | 305240 | 74 | 4590 | 66 | 1.12 |
| Disorder of cardiovascular system | 339240 | 82 | 5210 | 75 | 1.09 |
| Acute disease | 247350 | 60 | 3850 | 55 | 1.09 |
| Infectious disease | 258710 | 63 | 4070 | 58 | 1.09 |
| Disorder of gastrointestinal tract | 278390 | 68 | 4380 | 63 | 1.08 |
| Chronic disease | 225420 | 55 | 3630 | 52 | 1.06 |
| Disorder of abdomen | 315850 | 77 | 5120 | 73 | 1.05 |
| Disorder of head | 284740 | 69 | 4610 | 66 | 1.05 |
| Degenerative disorder | 290980 | 71 | 4760 | 68 | 1.04 |
| Disorder of digestive tract | 299900 | 73 | 4900 | 70 | 1.04 |
| Disorder of respiratory system | 299470 | 73 | 4860 | 70 | 1.04 |
| Disorder of abdominal segment of trunk | 325210 | 79 | 5310 | 76 | 1.04 |
| Disorder of digestive system | 309880 | 75 | 5110 | 73 | 1.03 |
| Disorder of trunk | 367110 | 89 | 6080 | 87 | 1.02 |
| Disorder of connective tissue | 283230 | 69 | 4740 | 68 | 1.01 |
| Disorder of digestive organ | 292850 | 71 | 4860 | 70 | 1.01 |
| Disorder of soft tissue | 348080 | 85 | 5880 | 84 | 1.01 |
| Disorder of skeletal system | 227020 | 55 | 3860 | 55 | 1 |
| Degenerative disorder of musculoskeletal system | 239650 | 58 | 4050 | 58 | 1 |
| Disorder of upper digestive tract | 252850 | 61 | 4240 | 61 | 1 |
| Osteoarthritis | 239630 | 58 | 4050 | 58 | 1 |
| Finding with explicit context | 282170 | 69 | 4890 | 70 | 0.99 |
| Soft tissue lesion | 263080 | 64 | 4500 | 65 | 0.98 |
| Musculoskeletal and connective tissue disorder | 252610 | 61 | 4360 | 63 | 0.97 |
| Disorder of extremity | 278390 | 68 | 5000 | 72 | 0.94 |
| Situation with explicit context | 321660 | 78 | 5800 | 83 | 0.94 |
| Disorder of lower extremity | 231190 | 56 | 4170 | 60 | 0.93 |
| Drug-related disorder | 276660 | 67 | 5090 | 73 | 0.92 |
| Procedure with explicit context | 228280 | 56 | 4330 | 62 | 0.9 |
| Allergic condition | 254770 | 62 | 4960 | 71 | 0.87 |
| Propensity to adverse reactions | 239310 | 58 | 4700 | 67 | 0.87 |
| Hypersensitivity disposition | 239300 | 58 | 4700 | 67 | 0.87 |
| Allergic disposition | 237880 | 58 | 4670 | 67 | 0.87 |
| Propensity to adverse reactions to substance | 235980 | 57 | 4630 | 66 | 0.86 |
| Allergy to substance | 234470 | 57 | 4600 | 66 | 0.86 |
| Hypersensitivity condition | 266210 | 65 | 5310 | 76 | 0.86 |

**Table S21**: Top 50 common clinical findings between adalimumab group and no-drug group

| Description | Count_No-drug | Proportion_No-drug (%) | Count_Adalimulab | Proportion_Adalimumab (%) | Proportion ratio |
| --- | --- | --- | --- | --- | --- |
| Finding of region of thorax | 321090 | 78 | 4900 | 70 | 1.11 |
| Finding of upper trunk | 328240 | 80 | 5090 | 73 | 1.1 |
| Urogenital finding | 289690 | 70 | 4480 | 64 | 1.09 |
| Cardiovascular finding | 342970 | 83 | 5290 | 76 | 1.09 |
| General body state finding | 247620 | 60 | 3910 | 56 | 1.07 |
| Gastrointestinal tract finding | 288940 | 70 | 4590 | 66 | 1.06 |
| Evaluation finding | 274780 | 67 | 4470 | 64 | 1.05 |
| Head finding | 295460 | 72 | 4790 | 69 | 1.04 |
| Abdominal organ finding | 314310 | 76 | 5090 | 73 | 1.04 |
| Respiratory finding | 314680 | 77 | 5160 | 74 | 1.04 |
| Viscus structure finding | 351300 | 85 | 5720 | 82 | 1.04 |
| Pain of truncal structure | 253970 | 62 | 4190 | 60 | 1.03 |
| Digestive system finding | 316090 | 77 | 5220 | 75 | 1.03 |
| Finding of abdomen | 326890 | 79 | 5350 | 77 | 1.03 |
| Finding of head and neck region | 332310 | 81 | 5500 | 79 | 1.03 |
| Finding of abdominal segment of trunk | 339240 | 82 | 5590 | 80 | 1.03 |
| Finding of back | 222640 | 54 | 3670 | 53 | 1.02 |
| General finding of observation of patient | 319980 | 78 | 5380 | 77 | 1.01 |
| Finding of trunk structure | 377930 | 92 | 6350 | 91 | 1.01 |
| Neurological finding | 339330 | 83 | 5770 | 83 | 1.00 |
| General finding of soft tissue | 351840 | 86 | 5960 | 86 | 1.00 |
| Finding of body region | 401260 | 98 | 6810 | 98 | 1.00 |
| Finding of pelvic region of trunk | 255860 | 62 | 4310 | 62 | 1.00 |
| Sensory nervous system finding | 332030 | 81 | 5730 | 82 | 0.99 |
| Pain / sensation finding | 330710 | 80 | 5710 | 82 | 0.98 |
| Pain | 330040 | 80 | 5700 | 82 | 0.98 |
| Pain finding at anatomical site | 324350 | 79 | 5620 | 81 | 0.98 |
| Finding of sensation by site | 326410 | 79 | 5660 | 81 | 0.98 |
| Finding of limb structure | 303430 | 74 | 5400 | 77 | 0.96 |
| Administrative statuses | 270640 | 66 | 4890 | 70 | 0.94 |
| Finding of lower limb | 253840 | 62 | 4600 | 66 | 0.94 |
| Clinical history and observation findings | 369650 | 90 | 6660 | 96 | 0.94 |
| Integumentary system finding | 244050 | 59 | 4370 | 63 | 0.94 |
| Prevention status | 242020 | 59 | 4440 | 64 | 0.92 |
| Musculoskeletal pain | 216080 | 53 | 4010 | 58 | 0.91 |
| Skin AND/OR mucosa finding | 242010 | 59 | 4530 | 65 | 0.91 |
| Skin finding | 218530 | 53 | 4110 | 59 | 0.9 |
| Mental state, behavior and/or psychosocial function finding | 315240 | 77 | 6210 | 89 | 0.87 |
| Non-smoker | 241980 | 59 | 5050 | 72 | 0.82 |
| Health-related behavior finding | 284860 | 69 | 6000 | 86 | 0.8 |
| Behavior finding | 285480 | 69 | 6000 | 86 | 0.8 |
| Finding of tobacco smoking behavior | 264430 | 64 | 5580 | 80 | 0.8 |
| Finding of tobacco use and exposure | 278430 | 68 | 5940 | 85 | 0.8 |

**Table S22**: Top 50 common diagnoses between infliximab group and no-drug group

| Description | Count No-drug | Proportion No-drug (%) | Count Infliximab | Proportion Infliximab (%) | Proportion ratio |
| --- | --- | --- | --- | --- | --- |
| Traumatic and/or non-traumatic injury of anatomical site | 250150 | 61 | 2110 | 56 | 1.09 |
| Hypertensive disorder, systemic arterial | 277190 | 67 | 2340 | 62 | 1.08 |
| Disorder of blood vessel | 246470 | 60 | 2100 | 56 | 1.07 |
| Traumatic AND/OR non-traumatic injury | 260290 | 63 | 2230 | 59 | 1.07 |
| Disorder of the genitourinary system | 261830 | 64 | 2270 | 60 | 1.07 |
| Metabolic disease | 301360 | 73 | 2620 | 69 | 1.06 |
| Disorder of thorax | 300150 | 73 | 2620 | 69 | 1.06 |
| Acute disease | 247350 | 60 | 2160 | 57 | 1.05 |
| Disorder of thoracic segment of trunk | 305240 | 74 | 2690 | 71 | 1.04 |
| Disorder of cardiovascular system | 339240 | 82 | 2970 | 79 | 1.04 |
| Essential hypertension | 257390 | 63 | 2290 | 61 | 1.03 |
| Infectious disease | 258710 | 63 | 2320 | 62 | 1.02 |
| Disorder of head | 284740 | 69 | 2560 | 68 | 1.01 |
| Disorder of soft tissue | 348080 | 85 | 3210 | 85 | 1.00 |
| Disorder of respiratory system | 299470 | 73 | 2740 | 73 | 1.00 |
| Disorder of trunk | 367110 | 89 | 3400 | 90 | 0.99 |
| Disorder of abdomen | 315850 | 77 | 2990 | 79 | 0.97 |
| Finding with explicit context | 282170 | 69 | 2660 | 71 | 0.97 |
| Disorder of gastrointestinal tract | 278390 | 68 | 2650 | 70 | 0.97 |
| Disorder of abdominal segment of trunk | 325210 | 79 | 3080 | 82 | 0.96 |
| Disorder of digestive tract | 299900 | 73 | 2870 | 76 | 0.96 |
| Degenerative disorder | 290980 | 71 | 2800 | 74 | 0.96 |
| Soft tissue lesion | 263080 | 64 | 2540 | 67 | 0.96 |
| Disorder of digestive system | 309880 | 75 | 2970 | 79 | 0.95 |
| Chronic disease | 225420 | 55 | 2180 | 58 | 0.95 |
| Disorder of digestive organ | 292850 | 71 | 2840 | 75 | 0.95 |
| Disorder of connective tissue | 283230 | 69 | 2760 | 73 | 0.95 |
| Disorder of extremity | 278390 | 68 | 2720 | 72 | 0.94 |
| Situation with explicit context | 321660 | 78 | 3130 | 83 | 0.94 |
| Disorder of upper digestive tract | 252850 | 61 | 2490 | 66 | 0.92 |
| Osteoarthritis | 239630 | 58 | 2360 | 63 | 0.92 |
| Degenerative disorder of musculoskeletal system | 239650 | 58 | 2360 | 63 | 0.92 |
| Disorder of lower extremity | 231190 | 56 | 2300 | 61 | 0.92 |
| Drug-related disorder | 276660 | 67 | 2790 | 74 | 0.91 |
| Musculoskeletal and connective tissue disorder | 252610 | 61 | 2550 | 68 | 0.9 |
| Procedure with explicit context | 228280 | 56 | 2380 | 63 | 0.89 |
| Disorder of skeletal system | 227020 | 55 | 2370 | 63 | 0.87 |
| Allergic condition | 254770 | 62 | 2710 | 72 | 0.86 |
| Allergic disposition | 237880 | 58 | 2580 | 68 | 0.85 |
| Allergy to substance | 234470 | 57 | 2540 | 67 | 0.85 |
| Hypersensitivity condition | 266210 | 65 | 2900 | 77 | 0.84 |
| Propensity to adverse reactions | 239310 | 58 | 2590 | 69 | 0.84 |
| Hypersensitivity disposition | 239300 | 58 | 2590 | 69 | 0.84 |
| Propensity to adverse reactions to substance | 235980 | 57 | 2560 | 68 | 0.84 |

**Table S23**: Top 50 common clinical findings between infliximab group and no drug group

| Description | Count_No-drug | Proportion_No-drug (%) | Count_Infliximab | Proportion_Infliximab (%) | Proportion ratio |
| --- | --- | --- | --- | --- | --- |
| Blood vessel finding | 246880 | 60 | 2100 | 56 | 1.07 |
| Urinary system finding | 236310 | 57 | 2050 | 54 | 1.06 |
| Urogenital finding | 289690 | 70 | 2510 | 67 | 1.04 |
| Cardiovascular finding | 342970 | 83 | 3020 | 80 | 1.04 |
| Finding of region of thorax | 321090 | 78 | 2850 | 76 | 1.03 |
| Finding of upper trunk | 328240 | 80 | 2930 | 78 | 1.03 |
| General body state finding | 247620 | 60 | 2230 | 59 | 1.02 |
| Head finding | 295460 | 72 | 2660 | 71 | 1.01 |
| Neurological finding | 339330 | 83 | 3090 | 82 | 1.01 |
| Finding of trunk structure | 377930 | 92 | 3480 | 92 | 1.00 |
| General finding of soft tissue | 351840 | 86 | 3240 | 86 | 1.00 |
| Finding of head and neck region | 332310 | 81 | 3040 | 81 | 1.00 |
| Sensory nervous system finding | 332030 | 81 | 3070 | 81 | 1.00 |
| Respiratory finding | 314680 | 77 | 2890 | 77 | 1.00 |
| Finding of body region | 401260 | 98 | 3680 | 98 | 1.00 |
| Measurement finding | 228340 | 56 | 2100 | 56 | 1.00 |
| Finding of pelvic region of trunk | 255860 | 62 | 2350 | 62 | 1.00 |
| Pain / sensation finding | 330710 | 80 | 3050 | 81 | 0.99 |
| Pain | 330040 | 80 | 3050 | 81 | 0.99 |
| Pain finding at anatomical site | 324350 | 79 | 3000 | 80 | 0.99 |
| Finding of sensation by site | 326410 | 79 | 3020 | 80 | 0.99 |
| General finding of observation of patient | 319980 | 78 | 2990 | 79 | 0.99 |
| Evaluation finding | 274780 | 67 | 2570 | 68 | 0.99 |
| Pain of truncal structure | 253970 | 62 | 2390 | 63 | 0.98 |
| Measurement finding outside reference range | 223930 | 54 | 2070 | 55 | 0.98 |
| Finding of abdominal segment of trunk | 339240 | 82 | 3180 | 84 | 0.98 |
| Finding of abdomen | 326890 | 79 | 3060 | 81 | 0.98 |
| Clinical history and observation findings | 369650 | 90 | 3520 | 93 | 0.97 |
| Viscus structure finding | 351300 | 85 | 3300 | 88 | 0.97 |
| Musculoskeletal pain | 216080 | 53 | 2070 | 55 | 0.96 |
| Digestive system finding | 316090 | 77 | 3000 | 80 | 0.96 |
| Abdominal organ finding | 314310 | 76 | 2970 | 79 | 0.96 |
| Gastrointestinal tract finding | 288940 | 70 | 2740 | 73 | 0.96 |
| Finding of limb structure | 303430 | 74 | 2930 | 78 | 0.95 |
| Finding of back | 222640 | 54 | 2140 | 57 | 0.95 |
| Finding of lower limb | 253840 | 62 | 2500 | 66 | 0.94 |
| Integumentary system finding | 244050 | 59 | 2360 | 63 | 0.94 |
| Administrative statuses | 270640 | 66 | 2660 | 71 | 0.93 |
| Skin AND/OR mucosa finding | 242010 | 59 | 2420 | 64 | 0.92 |
| Prevention status | 242020 | 59 | 2400 | 64 | 0.92 |
| Mental state, behavior and/or psychosocial function finding | 315240 | 77 | 3170 | 84 | 0.92 |
| Skin finding | 218530 | 53 | 2210 | 59 | 0.9 |
| Behavior finding | 285480 | 69 | 2980 | 79 | 0.87 |
| Health-related behavior finding | 284860 | 69 | 2980 | 79 | 0.87 |
| Finding of tobacco use and exposure | 278430 | 68 | 2930 | 78 | 0.87 |
| Non-smoker | 241980 | 59 | 2610 | 69 | 0.86 |
| Finding of tobacco smoking behavior | 264430 | 64 | 2810 | 75 | 0.85 |

**Table S24**: Top 50 common diagnoses between methotrexate group and no-drug group

| Description | Count No-drug | Proportion No-drug (%) | Count Methotrexate | Proportion Methotrexate (%) | Proportion ratio |
| --- | --- | --- | --- | --- | --- |
| Disorder of blood vessel | 246470 | 60 | 49730 | 57 | 1.05 |
| Disorder of the genitourinary system | 261830 | 64 | 52520 | 61 | 1.05 |
| Disorder of gastrointestinal tract | 278390 | 68 | 56440 | 65 | 1.05 |
| Disorder of nervous system | 244820 | 60 | 50180 | 58 | 1.03 |
| Traumatic and/or non-traumatic injury of anatomical site | 250150 | 61 | 51130 | 59 | 1.03 |
| Traumatic AND/OR non-traumatic injury | 260290 | 63 | 53440 | 62 | 1.02 |
| Hypertensive disorder, systemic arterial | 277190 | 67 | 56890 | 66 | 1.02 |
| Disorder of digestive tract | 299900 | 73 | 62250 | 72 | 1.01 |
| Metabolic disease | 301360 | 73 | 62630 | 72 | 1.01 |
| Disorder of thorax | 300150 | 73 | 61960 | 72 | 1.01 |
| Disorder of thoracic segment of trunk | 305240 | 74 | 63060 | 73 | 1.01 |
| Disorder of digestive system | 309880 | 75 | 64240 | 74 | 1.01 |
| Disorder of abdomen | 315850 | 77 | 65680 | 76 | 1.01 |
| Infectious disease | 258710 | 63 | 54140 | 63 | 1.00 |
| Disorder of abdominal segment of trunk | 325210 | 79 | 68020 | 79 | 1.00 |
| Disorder of cardiovascular system | 339240 | 82 | 70960 | 82 | 1.00 |
| Disorder of digestive organ | 292850 | 71 | 61330 | 71 | 1.00 |
| Disorder of respiratory system | 299470 | 73 | 63050 | 73 | 1.00 |
| Disorder of trunk | 367110 | 89 | 77570 | 90 | 0.99 |
| Acute disease | 247350 | 60 | 53200 | 61 | 0.98 |
| Disorder of soft tissue | 348080 | 85 | 75270 | 87 | 0.98 |
| Essential hypertension | 257390 | 63 | 56230 | 65 | 0.97 |
| Disorder of upper digestive tract | 252850 | 61 | 54400 | 63 | 0.97 |
| Disorder of head | 284740 | 69 | 62440 | 72 | 0.96 |
| Finding with explicit context | 282170 | 69 | 63410 | 73 | 0.95 |
| Disorder of connective tissue | 283230 | 69 | 63700 | 74 | 0.93 |
| Drug-related disorder | 276660 | 67 | 62380 | 72 | 0.93 |
| Soft tissue lesion | 263080 | 64 | 59420 | 69 | 0.93 |
| Degenerative disorder | 290980 | 71 | 67060 | 77 | 0.92 |
| Situation with explicit context | 321660 | 78 | 74700 | 86 | 0.91 |
| Musculoskeletal and connective tissue disorder | 252610 | 61 | 59210 | 68 | 0.9 |
| Disorder of extremity | 278390 | 68 | 65640 | 76 | 0.89 |
| Allergic disposition | 237880 | 58 | 56380 | 65 | 0.89 |
| Allergy to substance | 234470 | 57 | 55480 | 64 | 0.89 |
| Hypersensitivity condition | 266210 | 65 | 63510 | 73 | 0.89 |
| Disorder of lower extremity | 231190 | 56 | 54910 | 63 | 0.89 |
| Disorder of skeletal system | 227020 | 55 | 53370 | 62 | 0.89 |
| Allergic condition | 254770 | 62 | 60450 | 70 | 0.89 |
| Propensity to adverse reactions | 239310 | 58 | 56820 | 66 | 0.88 |
| Hypersensitivity disposition | 239300 | 58 | 56820 | 66 | 0.88 |
| Propensity to adverse reactions to substance | 235980 | 57 | 55960 | 65 | 0.88 |
| Degenerative disorder of musculoskeletal system | 239650 | 58 | 57620 | 67 | 0.87 |
| Osteoarthritis | 239630 | 58 | 57610 | 67 | 0.87 |
| Procedure with explicit context | 228280 | 56 | 58880 | 68 | 0.82 |

**Table S25:** Top 50 common clinical findings between methotrexate group and no-drug group

| Description | Count No-drug | Proportion No-drug (%) | Count Methotrexate | Proportion Methotrexate (%) | Proportion ratio |
| --- | --- | --- | --- | --- | --- |
| Blood vessel finding | 246880 | 60 | 49830 | 58 | 1.03 |
| Gastrointestinal tract finding | 288940 | 70 | 58960 | 68 | 1.03 |
| Abdominal organ finding | 314310 | 76 | 65330 | 75 | 1.01 |
| Digestive system finding | 316090 | 77 | 65720 | 76 | 1.01 |
| Finding of region of thorax | 321090 | 78 | 66790 | 77 | 1.01 |
| Finding of upper trunk | 328240 | 80 | 68460 | 79 | 1.01 |
| Finding of body region | 401260 | 98 | 85010 | 98 | 1.00 |
| Cardiovascular finding | 342970 | 83 | 71840 | 83 | 1.00 |
| Respiratory finding | 314680 | 77 | 66680 | 77 | 1.00 |
| Urogenital finding | 289690 | 70 | 60570 | 70 | 1.00 |
| Finding of abdominal segment of trunk | 339240 | 82 | 71200 | 82 | 1.00 |
| Finding of abdomen | 326890 | 79 | 68190 | 79 | 1.00 |
| Finding of trunk structure | 377930 | 92 | 80020 | 92 | 1.00 |
| Viscus structure finding | 351300 | 85 | 73760 | 85 | 1.00 |
| Pain of truncal structure | 253970 | 62 | 54300 | 63 | 0.98 |
| General finding of soft tissue | 351840 | 86 | 76020 | 88 | 0.98 |
| Neurological finding | 339330 | 83 | 73450 | 85 | 0.98 |
| Finding of head and neck region | 332310 | 81 | 72080 | 83 | 0.98 |
| Head finding | 295460 | 72 | 64370 | 74 | 0.97 |
| General body state finding | 247620 | 60 | 53640 | 62 | 0.97 |
| Measurement finding | 228340 | 56 | 50500 | 58 | 0.97 |
| Sensory nervous system finding | 332030 | 81 | 73020 | 84 | 0.96 |
| Pain / sensation finding | 330710 | 80 | 72780 | 84 | 0.95 |
| Pain | 330040 | 80 | 72580 | 84 | 0.95 |
| Finding of sensation by site | 326410 | 79 | 72140 | 83 | 0.95 |
| Pain finding at anatomical site | 324350 | 79 | 71720 | 83 | 0.95 |
| General finding of observation of patient | 319980 | 78 | 70640 | 82 | 0.95 |
| Measurement finding outside reference range | 223930 | 54 | 49590 | 57 | 0.95 |
| Evaluation finding | 274780 | 67 | 61630 | 71 | 0.94 |
| Finding of pelvic region of trunk | 255860 | 62 | 56740 | 66 | 0.94 |
| Clinical history and observation findings | 369650 | 90 | 83150 | 96 | 0.94 |
| Integumentary system finding | 244050 | 59 | 55370 | 64 | 0.92 |
| Finding of limb structure | 303430 | 74 | 70470 | 81 | 0.91 |
| Finding of lower limb | 253840 | 62 | 60140 | 69 | 0.9 |
| Administrative statuses | 270640 | 66 | 64450 | 74 | 0.89 |
| Skin finding | 218530 | 53 | 52100 | 60 | 0.88 |
| Skin AND/OR mucosa finding | 242010 | 59 | 57710 | 67 | 0.88 |
| Mental state, behavior and/or psychosocial function finding | 315240 | 77 | 77100 | 89 | 0.87 |
| Prevention status | 242020 | 59 | 59600 | 69 | 0.86 |
| Musculoskeletal pain | 216080 | 53 | 54140 | 63 | 0.84 |
| Behavior finding | 285480 | 69 | 73720 | 85 | 0.81 |
| Health-related behavior finding | 284860 | 69 | 73620 | 85 | 0.81 |
| Finding of tobacco use and exposure | 278430 | 68 | 72780 | 84 | 0.81 |
| Finding of tobacco smoking behavior | 264430 | 64 | 69170 | 80 | 0.8 |
| Non-smoker | 241980 | 59 | 64200 | 74 | 0.8 |
